# Nurse Practitioners’ Practice Patterns in Primary Care in Finland: A Descriptive Qualitative Study

**DOI:** 10.1177/23333936251365612

**Published:** 2025-08-18

**Authors:** Ruwang Han, Melanie Rogers, Sofia Asplund, Lisbeth Fagerström

**Affiliations:** 1 Åbo Akademi University, Vaasa, Finland; 2 University of Huddersfield, UK

**Keywords:** advanced practice nursing, qualitative research, primary care, practice patterns, nurse practitioner, Finland

## Abstract

Healthcare demands continue to increase across the globe, Advanced Practice Nurses (APNs) including Nurse Practitioners (NPs) are recognised as a valuable workforce that can increase access and improve the quality of care. However, NPs are underrepresented in Finland. This study explored Finnish NPs’ practice patterns and identified the common visit types to NP clinics in primary care. This descriptive qualitative study is part of a multiple-method and action research project that aims to develop and evaluate advanced practice nursing models in primary care within a wellbeing services county of Western Finland. Responses to open-ended questions and interview data from 16 NPs working in primary care were analysed using inductive content analysis. The findings highlight key aspects of NPs’ work, including the types of patients they care for—those with both acute and long-term health needs—and the core elements of their practice, such as conducting health assessments, providing counselling and coaching, engaging in collaborative care, and prescribing medications. The analysis also revealed persistent ambiguity surrounding certain aspects of NP practice. Overall, the findings indicate that NPs in Finland utilise a wide range of knowledge and skills to care for patients presenting with acute and long-term health problems.

## Introduction

Healthcare demands in primary care continue to increase, with countries across the globe struggling to strengthen their workforces to improve access for all patients (Maier et al., 2017). The World Health Organization (WHO, 2025) defines primary care as a model of care that supports first-contact, accessible, continuous, comprehensive, and coordinated person-focused care. Internationally, the COVID-19 pandemic increased the urgency to address longstanding pressures on health systems, highlighting the need for improved access and treatment in primary care. This became more apparent in light of the acute shortage of primary care physicians (Brownwood & Lafortune, 2024).

In Finland, long waiting times, an ageing population, multiple comorbidities, and polypharmacy have resulted in many patients facing unmet healthcare needs. In 2022, the extent of unmet healthcare needs in Finland was nearly three times higher than the European Union (EU) average, according to Organization for Economic Cooperation and Development (OECD, 2023). In OECD countries, the main reasons for expanding nurse roles include addressing primary care physician shortages, promoting better access to primary care, promoting continuity and quality of care, responding to cost containment pressures, and promoting nursing career progression and retention (Brownwood & Lafortune, 2024). Since Finland has fewer physicians per 1,000 population than the EU average (OECD, 2023), an exploration into how Advanced Practice Nurses (APNs) are utilised in primary care is important.

Globally, many APNs practice in primary care settings (Schober, 2023). The implementation of advanced practice nursing has often started in primary care or in rural areas. Many countries have developed advanced practice to its full scope, including the United States (US), Canada, the United Kingdom (UK), and Australia (Spies et al., 2024). Nevertheless, advanced practice nursing in primary care is still a new concept in many countries (Fagerström, 2021).

### Advanced Practice Nursing from a Global Perspective

Advanced practice nursing is evident in over 80 countries (Miller et al., 2024). The International Council of Nurses (ICN) defines an APN as:

A generalist or specialised nurse who has acquired, through additional graduate education (minimum of a master’s degree), the expert knowledge base, complex decision-making skills, and clinical competencies for Advanced Nursing Practice, the characteristics of which are shaped by the context in which they are credentialed to practice. (ICN, 2020, p. 6)

The two most common APN roles globally are the Clinical Nurse Specialist (CNS) and the Nurse Practitioner (NP). NPs commonly work as generalists in primary care but are also found in all practice settings (Maier et al., 2017; Torrens et al., 2020). In countries that have fully implemented advanced practice nursing, increasing the number of APNs has been seen as one solution to respond to primary care needs and reduce pressures on primary care physicians and hospitals (Brownwood & Lafortune, 2024). Unsworth et al. (2024) suggest that, internationally, the successful implementation of APN roles can only be achieved through clear policy, regulation, and legislation. A national policy is regarded as an important facilitator in promoting advanced practice nursing models and roles in practice. However, ongoing challenges include resistance from medical professionals and disagreements among nursing leaders about implementing these roles, which sometimes result in APNs being unable to work within their full scope of practice (Maier et al., 2017; Unsworth et al., 2024).

The development of APN policies and regulations varies from country to country (Unsworth et al., 2024; Wheeler et al., 2024), and significant challenges have been reported globally, including variations in title, title protection, regulation, credentialing, and barriers to practice (Wheeler et al., 2022). Furthermore, APN roles are often recognised differently, whether by governmental regulations, professional organisations, or a lack of formal regulations (Scanlon et al., 2020). Overall, the lack of national policies, regulations, and legislation continues to obstruct progress in APN role development, for example, as seen in Kenya (Ndirangu-Mugo et al., 2024).

In Europe, a minimum educational requirement of a master’s degree for an APN has been established in national legislation in several countries, including Ireland and the Netherlands (De Raeve et al., 2024). However, many countries are still struggling to utilise the full potential of advanced practice nursing. In France, about 30% of APN respondents are still employed as Registered Nurses (RNs), despite the implementation of APN legislation and regulatory measures (Devictor et al., 2023). Although advanced practice nursing has been developed for many years in the UK, title protection and regulation of the practice are still lacking (Rogers & Gloster, 2020). Advanced practice nursing is still in its infancy in Finland, despite almost 20 years of education in the field and limited prescription rights since 2011, and the same applies to Norway, despite specialist accreditation being available since 2019 (Fagerström, 2021). In Norway, NPs do not have the prescriptive authority, public health nurses and midwives have limited prescribing authority (Norwegian Directorate of Health, 2024)

### The Development of Advanced Practice Nursing in Finland

In Finland, advanced practice nursing roles include CNSs, NPs, and nurse prescribers. Although the education for nurse prescribing is not a master’s degree in Finland, nurse prescribers acquire the clinical skills necessary to practice at an advanced level (Suutarla et al., 2023). Current estimates of APNs in Finland include 100 CNSs, 300–400 NPs (WHO, 2023) and 691 nurse prescribers (Suutarla et al., 2023). In Finland, CNSs usually work in specialised care (e.g. palliative care, cardiovascular care, neurological care, and nephrological care) and hospital settings, while NPs and nurse prescribers primarily work in primary care settings (Finnish Nurse Association [FNA], 2023).

Generally, NPs are utilised in primary care despite issues related to title protection and regulation (Suutarla et al., 2023). Finland’s first NP education programme in Swedish was developed at Novia University of Applied Sciences (UAS) in 2006 (Fagerström, 2021), and after that, several other Finnish UASs started providing theoretical NP education at 90 credits. In 2021, Åbo Akademi University began delivering an international APN master’s programme consisting of a combination of theory and practice (120 credits). A total of 450 practice hours is mandatory, and the programme follows ICN international recommendations (FNA, 2023). The graduates of this programme have advanced clinical competence skills and the capability to work as NPs. However, due to job descriptions and titles vary in Finland, there is no data regarding the number of NPs (FNA, 2023; Jokiniemi et al., 2022).

Some Finnish NPs also have limited prescriptive authority. Nurse prescribing, introduced in Finland in 2011, is fundamental to the NP role. National programmes for nurse prescribing are taught separately from the NP curriculum and as a standalone postgraduate qualification (45 credits). RNs who pass the national nurse prescribing programme have limited prescriptive authority (Fagerström, 2021). Nurse prescribers in Finland are registered with the National Supervisory Authority for Welfare and Health (Valvira, n.d.). Among the challenges to developing NP roles in Finland are that not all NPs have prescriptive authority, and they are not allowed to diagnose diseases. However, NPs can assess patients who present with undifferentiated and undiagnosed conditions and conduct diagnostic reasoning but need a physician to confirm the diagnosis. Diagnosis is defined as “the art of identifying a disease by the signs, symptoms, and test results of a patient” (Beer et al., 2017, p. 45), while diagnostic reasoning is “the process to identify a hypothesis that best explains the clinical evidence” (Szaflarski, 1997). With nurse prescribing education, NPs are able to prescribe certain medications, including antibiotics, based on patients’ symptoms (Ministry of Social Affairs and Health [STM], 2010).

Primary care services are usually provided at healthcare centres across Finland (STM, 2024). In 2023, Finland implemented a national reform of its healthcare system, dividing the entire country into 21 Wellbeing Services Counties, which are responsible for organising healthcare, social welfare, and rescue services (STM, 2025). A Wellbeing Services County in Western Finland serves a bilingual (Finnish and Swedish) population of about 175,000. It was selected for this study due to its increasing APN development. The land area of this county is about 7,932.36 km^2^. In 2023, an action research collaboration started between the university and the Wellbeing Services County, including researchers and healthcare managers.

Despite two decades of developing NP education and nurse prescribing in Finland, there is still limited evidence regarding NPs’ practice. Jokiniemi et al. (2022) have pointed out the role distinction of CNSs in specialist and hospital contexts and other generalist and specialist nursing roles in Finland, and the CNSs’ job description is clearly presented (FNA, 2023). However, Finnish NPs working in primary care are underrepresented. Therefore, this study aimed to identify common visit types to NP clinics and explore the practice patterns of NPs working in primary care in Finland.

## Methods

### Research Design

A qualitative description approach, as described by Sandelowski (2010), was employed to explore Finnish NPs’ practice in primary care. Qualitative descriptive studies are characterised by their close adherence to the data and to the surface meaning of words and events (Sandelowski, 2000). This approach is distinct from other qualitative approaches, since it represents a less interpretative form of qualitative research (Sandelowski, 2000, 2010). Qualitative descriptive research is grounded in naturalistic inquiry (Bradshaw et al., 2017; Lincoln & Guba, 1985), as it seeks to represent phenomena within their natural context. This approach, therefore, can be used to enable researchers to gain insight into the daily work and practices of Finnish NPs through participants’ descriptions of their experiences. The hallmarks of qualitative description include using straightforward approaches to data collection and analysis, emphasising low-inference interpretation, and reporting the findings that focus on comprehensive and detailed descriptions of participants’ experiences in their own words (Bradshaw et al., 2017; Sandelowski, 2000, 2010).

This study was inspired by the three-dimensional view of knowledge as a synthesis of knowledge, skills, and wisdom, and the Caring Advanced Practice Nursing model (Fagerström, 2021). The three-dimensional view of knowledge is rooted in Aristotle’s (2014; ca. 350 B.C.E.) philosophy, which identified different forms of knowledge: episteme, teckne, and phronesis. Gustavsson (2000) expanded this idea to include theoretical-scientific knowledge, practical skills, and practical wisdom, highlighting the role of context. Moreover, Molander (1996) defined this concept as “knowledge in action,” and this study explored how NPs utilised their knowledge in practice within primary care settings.

Furthermore, this study is part of the first phase of a multiple-method and action research project that focuses on the development and evaluation of advanced practice nursing models in primary care in Western Finland. The action research consists of three phases: (1) describe the scope of practice for APNs, including NPs’ practice patterns, and identify patient needs in primary care using multiple methods; (2) explore patient satisfaction with operative advanced practice nursing models (including NPs and nurse prescribers) in primary care through a quantitative approach; (3) conduct a cost analysis of operative advanced practice nursing models in primary care using a quantitative method. Further phases will focus on evaluating the outcomes of operative advanced practice nursing models in primary care. This study provides insights into NPs’ practice patterns and common visit types to NP clinics in primary care, offering evidence that will also inform further studies, including patient experiences of APN consultations and the effectiveness of APN roles. The findings were reported according to the consolidated criteria for reporting qualitative research (COREQ) checklist (Tong et al., 2007).

### Recruitment and Participants

Recruitment was enabled through a list of APNs working in the county developed by the initial project team. In terms of purposeful sampling (Sandelowski, 2000), all participants had NP work experience in primary care within a wellbeing services county in Western Finland, and they represented a diverse range of work areas. The inclusion criteria for participants included an RN with NP education (90 credits or 120 credits), with or without prescription rights, and currently or recently working in an NP role in primary care. All participants were contacted by e-mail and invited to participate in the study. In this county, a total of 18 NPs (nine with prescription authority and nine without) working in primary care were invited to participate in the study, of whom 16 agreed (*N* = 15 Swedish speaking, *N* = 1 Finnish speaking). Two of the 16 participants did not complete the questionnaire with open-ended questions; however, they were willing to be interviewed. These data were included in the analysis. Most of the participants (*N* = 14) worked clinically as NPs. Two participants trained as NPs worked in areas where their role primarily involved administrative tasks; one continued to care for patients, while the other had several years of experience as an NP in primary care.

### Data Collection

Semi-structured interviews and questionnaires were used to collect qualitative data. In qualitative descriptive studies, data collection is usually directly discovering about “who,” “what,” and “where” of events or experiences (Sandelowski, 2000). This study collected qualitative data in the first phase of the project through multiple methods (Morse, 2003) relevant to the study’s aim. Interviews were conducted with participants at their workplaces (*N* = 7) and at the university (*N* = 1) between May and December 2023. The first, third, and fourth authors (R.H., S.A. & L.F.) and one research assistant conducted the interviews (in Finnish, *N* = 1; in Swedish, *N* = 7) in the following styles: six dyadic interviews (two participants in a group), one triadic interview (three participants in a group), and one individual interview, due to practical reasons, such as different workplaces, time constraints, and long geographical distances. Interview dyads or triads can be used to explore complex complementary and multiple perspectives (Kendall et al., 2009). All interviews were conducted face-to-face (*N* = 7) or through a video conference meeting (*N* = 1) using a semi-structured interview guide that aimed to explore NPs’ experiences and practice patterns. A video conference interview was conducted due to distance and the participants’ work schedules. All interviews were conducted in a quiet room and only included the researchers and participants. The video conference offers convenience, and participants can attend anytime and anywhere, while it is easier to observe whole-body language during face-to-face interviews. Both face-to-face meetings and video conferences were conducted similarly, and this did not affect data collection.

Two main questions were included in the interview guide: (1) Please describe your work experiences as an NP (i.e. please describe your areas of responsibility at work, including the patient groups you serve and the care activities you conduct); and (2) Please describe your work tasks as an NP (i.e. whether there is a written job description for the role of NPs; please describe your practice patterns, work tasks, and responsibilities). The interview guide was pilot tested, and no amendments were necessary. Field notes were taken during the interviews, and reflections were written after each interview. The interviews were recorded and transcribed verbatim. The length of the interviews varied between 25 and 57 min; the average duration was 41 min.

At the beginning of the interviews, participants were asked to fill in a shortened version of the Finnish Nurse Prescribing Questionnaires (FinNPQ; Fagerström et al., 2023), which included background information and 31 statements about care activities regarding the eight APN core competencies (direct clinical care, consultation, cooperation, case management, coaching and guidance, research and development, ethical decision-making, and leadership), according to the Caring Advanced Practice Nursing Model (Fagerström, 2021). The first core competency of direct clinical care was assessed with the following four open-ended questions:

(1) Please describe the patient groups for which you provide care and are responsible.
(2) Please briefly describe the most common health problems and symptoms of your patients at work.
(3) Please describe the commonly used care activities and care measures in your role.
(4) Please describe your work tasks related to health promotion and disease prevention.

Background information and answers to these four open-ended questions were used in this study. The data from the entire shortened version of the FinNPQ will be reported in another study on APNs’ core competencies. Both Finnish and Swedish versions were language checked by bilingual researchers in the research group.

### Ethical Considerations

This study followed research guidelines published by the Finnish National Board of Research Integrity (TENK, 2023) and the General Data Protection Regulation (GDPR; EU, 2016). The research permit included ethical approval obtained from the regional wellbeing services county on May 23, 2023 (registration number: 681/13.01/2023). The participants were given written and verbal information about the research and how the data and personal information would be used and protected, including its use for publications. To protect the anonymity of the participants, they were instructed not to share information about their participation during the interviews. All participants gave written consent, and their participation was voluntary and confidential. Participants had the right to request that their data not be used at any time. To avoid identifying participants in the results, their names were coded into letters of the alphabet and numbers. Only the first and fourth authors (R.H. & L.F.) could see the final version of the name list with these codes.

#### Data Analysis and Synthesis

Data from the four open-ended questions from the FinNPQ questionnaire and the interviews were analysed using inductive content analysis undertaken within NVivo software (version 14, 2024). Qualitative content analysis is suitable for qualitative descriptive studies (Sandelowski, 2000). The process of data analysis consisted of data reduction, data grouping, and the formation of concepts (Kyngäs et al., 2019), as the categories were derived directly from the data. The transcripts were read several times and checked by returning to the recordings. The responses to the open-ended questions and the transcripts were analysed separately. The responses to the open-ended questions provided additional details to support the results from the interviews. All authors participated in the data analysis by creating open codes and discerning the subcategories, and all subcategories and categories were discussed several times to ensure agreement. A total of 791 codes were initially identified, and open codes were grouped by comparing content similarities and differences. In the data abstraction phase, open codes formed subcategories (Kyngäs et al., 2019). An example of this process is shown in Table 1. Coding trees were created in NVivo with the hierarchy “open codes,” “subcategories,” and “categories.” With the integration of all codes, they were grouped into six subcategories and three categories. Quotations were translated from Swedish and Finnish into English.

**Table 1.** Example of Inductive Content Analysis.

Original text or answer	Codes	Subcategory	Category
“I consult physicians when I need to ask them for antibiotic prescriptions.” (R1)	Consultation for prescribing medicines	Collaborative approaches to practice	Main features of NPs’ practice patterns
“We can consult cardiac nurses regarding our patients.” (R5)	Ask specialist nurses in the hospital		
“We have excellent collaboration with the pharmacy. The patient will be informed about when to withdraw from medications and renew her/his prescriptions. Then, the patient will contact us if there is a need for [follow-up] appointments . . . it has worked very smoothly in this way.” (R16)	Collaboration with the pharmacy		
“We contact other colleagues when necessary, [instead of] just sitting in our rooms thinking alone.” (R6)	Cooperation with others		
[I] consulted physicians [about my patients] (R11)	Consultation		

*Note*. NP = nurse practitioner; R = respondent.

### Rigour and Trustworthiness

Rigour is important for enhancing the consistency and quality of qualitative research (McBrien, 2008), and the trustworthiness of a qualitative content analysis usually refers to credibility, dependability, transferability, and confirmability (Lincoln & Guba, 1985). To ensure credibility, the data were collected using the participants’ native languages, Finnish and Swedish. The quotations were translated from Swedish and Finnish into English by R.H., and co-authors (S.A. and L.F.) conducted member-checking for accurate translations. One of the authors (M.R.), a native English speaker and NP, reviewed the English translations. Additional questions were sent to participants after the interviews to ensure that their practices were described accurately. A detailed audit trail was maintained by R.H. to document the data collection and analysis meetings, as well as all changes in this study, to enhance dependability. Regarding transferability, this study provides a detailed description of the context and sample, allowing readers to understand NPs’ practice patterns in similar contexts. In terms of confirmability, the codes created by NVivo were systematically checked by the author team. All authors participated in regular meetings and discussed the open codes, subcategories, and categories until agreement was reached. R.H. is a male RN in Finland and a PhD candidate. The other authors of this international team are female researchers and have extensive experience in qualitative studies.

## Results

The background characteristics of the participants are presented in Table 2. The participants were predominantly female; only one male NP was included in the study. Nearly half (44%) of the participants had prescriptive authority. All participants had over 12 years of clinical work experience in a variety of healthcare settings and had worked as NPs in primary care.

**Table 2.** Demographics (*N* = 16).

Characteristics	No.	Percentage
1. *Sex*		
Male	1	6
Female	15	94
2. *Nurse prescribing*		
Yes	7	44
No	9	56
3. *Current work areas*		
Acute care unit at the healthcare centre	8	50
NP clinic for chronic diseases	6	37
Administrative job and other	2	13
4. *Age (in years)*		
Under 45	7	44
45–55	4	25
Over 55	5	31
5. *Work experience as registered nurse, public health nurse, or midwife (in years)*		
12–19	6	37
20–29	7	44
Over 29	3	19
6. *NPs with special competence(s)*		
Children’s health care	1	6
Women’s health care	1	6
Refugee health care	1	6
None	13	82
7. *Work experience as NP (in years)*		
Less than 3	7	44
3–10	6	37
Over 10	3	19
8. *Working hours as NP*		
Less than 20%	2	13
20–80%	4	25
100%	10	62

*Note.* NP = nurse practitioner; *N* = number of participants; No. = number of responses.

The results from the content analysis are organised into three categories and six subcategories, focusing on key aspects of NP’s work (Table 3). The first category focuses on types of patients NPs cared for - those with both acute conditions and long-term health problems. Key features of NP practice make up the second category and include conducting health assessments, delivering counselling and coaching, collaborating with other healthcare providers, and prescribing medications. The third category describes persistent ambiguity in aspects of NP roles and responsibilities, suggesting variability in how their practice is understood and implemented. Participants are referred to as R1 to R16.

**Table 3.** Categories and Subcategories.

Categories	Subcategories
Common visit types to NP clinics	Acute care Long-term health care
The main features of NPs’ practice patterns	Health assessment Counselling and coaching Collaborative approaches to practice Nurse prescribing
Ambiguity in NPs’ practice patterns	

*Note.* NP = nurse practitioner.

### Category 1. Common Visit Types to NP Clinics

#### Acute Care

About half of the NPs saw patients with acute health problems who presented with undifferentiated or undiagnosed conditions. The NPs emphasised that they offer holistic assessments to patients across age ranges. One participant reflected on this point: “An NP views the patient as a whole human being” (R11). Another participant said, “We take care of patients among all age groups . . . The patient’s problem can be diffuse and multifaceted, which might require an appointment with a physician” (R1).

The most common acute health problems seen included pain, infections, dizziness, skin issues, and allergies. Gastrointestinal and musculoskeletal problems, as well as deterioration in diagnosed conditions, were also mentioned as common acute health problems.

#### Long-Term Health Care

Eight of the participants reported seeing patients with long-term conditions, mainly cardiovascular disease, respiratory disease, diabetes, renal disease, and cognitive decline. Instead of these patients seeing a physician, one NP clinic in the centre of the county and another in the south were responsible for follow-up consultations for long-term health problems. The NPs reflected that patients were given more time to discuss their concerns when seeing an NP, as they would listen to their problems. The participants emphasised the importance of holistic and bio-psycho-social approaches. As one NP mentioned, “When [patients] consult with NPs, they [would like] to tell their whole life story to the NPs” (R7). Another NP also said:

When an NP meets a diabetes patient, it is not just about addressing the disease but also about other aspects. The NP acts like a spider, working to ensure that the patient’s needs are met on all levels and that everything works well at home. (R12)

In addition, the participants reported that patients with long-term health problems often presented with multiple symptoms and/or complications related to polypharmacy and their declining health. The NPs were able to assess these and provide support and care by utilising their advanced skills and expertise.

### Category 2. Main Features of NPs’ Practice Patterns

#### Health Assessment

All of the NPs 1mentioned health assessment as the most frequent part of their clinical practice. Health assessment included clinical examination, history taking, and a review of medication before considering management. The NPs were able to order common laboratory tests and review these in relation to assessment and management. Several NPs mentioned that because of their additional training, they were more knowledgeable and able to conduct more comprehensive clinical assessments and examinations than RNs. Examples of the skills they reported include chest auscultation; ear, nose, and throat examination; and cardiovascular and abdominal examination. They were also skilled in health promotion and risk assessment. For instance, one participant said, “The level of an NP’s physical assessment is almost equivalent to a physician’s” (R13). Another participant noted, “We have complete freedom and responsibility, and request laboratory tests when necessary” (R5).

One NP specialising in elderly care conducted fall, nutrition, and memory assessments for the elderly during home visits. For NPs working with patients’ long-term health problems, a health assessment was included in the follow-up consultation. One NP reflected that their practice included “engaging in patients’ treatment interventions, reviewing their laboratory results, and reviewing their medications” (R11).

#### Counselling and Coaching

Counselling and coaching constitute a significant part of the NPs’ work with patients presenting with both acute and long-term health problems. The NPs reported that patients often seek advice when starting a new treatment and that consultations include patients across all age groups with diverse needs. The NPs usually counselled patients directly, but some also spent time with carers or relatives in need of coaching, providing services such as teaching, supervision, and giving advice on health promotion and disease prevention. One NP commented, “I take care of prediabetic patients’ first visits and give information about diet, motion, weight loss, and smoking” (R12). Meanwhile, another NP stated: “We always make sure to inform patients that they can reach out to us directly whenever necessary” (R16).

Patients with new diagnoses often need more frequent coaching. This may include repeating information about medications and monitoring blood results. For instance, one NP mentioned, “[I] supervise patients on how they use their medications” (R14). The NPs also engaged in supportive and motivational dialogue with patients and guided them in their lifestyle choices, such as weight control, exercising, and dieting. One NP was responsible for infection group education and participated in a wound care advisory group.

#### Collaborative Approaches to Practice

The NPs collaborated with not only physicians and coworkers but also other professionals, such as pharmacists, social workers, rehabilitation teams for patients living at home, and employment and economic organisations for unemployed patients. The most common collaboration was between NPs and physicians; this could occur physically or remotely, depending on the workplace. For some NPs, this was daily or weekly. They mentioned that positive collaboration with a supervising physician was most likely to help them develop their scope of practice. Several NPs mentioned that physicians appreciated consulting with the NPs, as they suggested that this was time saving for them. One participant shared that “the collaboration between the physician and I works well. Since we work together closely and intensively, we get to know each other and see each other’s weaknesses and strengths” (R10). Another participant added:

We know [which physicians] we can ask for help, those who know NPs [roles], and how they work . . . But if it is a new physician, there could be a problem of consultation between him/her and the NP because he/she might not know what knowledge NPs have. (R8)

Furthermore, some NPs reported that physicians, medical students, and RNs consulted them about specific patients under their care, as they understood the level of knowledge the NPs had about the patients in their care. It was seen as an obstacle to practice if the physicians collaborating with the NPs did not understand the competencies of NPs.

#### Nurse Prescribing

All participants who had the right to prescribe medications (*N* = 7) mentioned that this was one of the core practice patterns needed to attain the full NP scope of practice. These seven participants were able to change medications and carry out monitoring investigations according to national guidelines. On the one hand, this practice occurred more often in acute care settings. On the other hand, one NP working with long-term health problems said, “We manage follow-up independently and change medications according to guidelines. If we change one medication, we check which lab tests are needed” (R5).

As a result, one NP mentioned that patients were satisfied with their visits to NPs because they could carry out investigations and prescribe at the same consultation. However, all NPs with prescribing authority expressed that the formulary they are legally allowed to prescribe from is too limited, as it is restricted to specific ages and diagnoses; for example, antibiotics can be prescribed only for urinary tract infections (UTIs), pharyngitis, breast inflammation, and eye infections, based on the patients’ symptoms. As one participant stated, “We take care of many patients with ear infections . . . so, [from my point of view], the scope of nurse prescribing could be extended there” (R8). One challenge that NPs face in Finland is regarding prescriptive authority, as they are not allowed to diagnose diseases, despite their skills in diagnostic reasoning.

### Category 3. Ambiguity in NPs’ Practice Patterns

Although some participants described the types of health problems they encountered and how their skills were utilised at work, a lack of clarity in their roles and scope of practice was identified as a barrier to the effective implementation of advanced practice nursing models. Only five participants had written job descriptions. All participants valued their NP education and skills, and they also expressed that their NP roles were meaningful. Still, they experienced that their practice patterns were unclear, and their roles were invisible in the current health care system:

I work with [good and experienced nurses], and we [take care of] all patients. When a new patient comes to us, it just depends on who has free time [to see the patient]. But I would prefer that those patients be directed to me in certain things. (R2)

Due to unclear practice patterns, NPs reported spending their work time on tasks that did not utilise their competencies. As a result, some participants expressed frustration regarding unclear practice patterns:

[I] would like to have more time for follow-up consultations with patients suffering from chronic diseases, [allowing me] to care for them peacefully. And I do not need to spend time on other work tasks [which are not related to NPs’ practice patterns] all the time. (R5)

I also see that NPs could have more of a consultative and perhaps leading role. Since an NP has a master’s degree, he or she could have that consulting, leading, and supervising function . . . that competence has not been [fully] utilised. (R1)

The NPs stated that they received support from colleagues and nurse managers in their workplaces. However, more than half (*N* = 10) mentioned that the organisation’s leaders were unaware of, and did not understand, the NP’s role and competencies. Therefore, they identified a need for more organisational support to clarify NPs’ practice patterns, as in the form of a role description.

## Discussion

In this study, NPs commonly consulted patients with acute and/or long-term health problems. Within their clinical practice in primary care, they utilised health assessment, counselling and coaching, and collaborative approaches to practice and nurse prescribing. Despite the role being expanded, many of the NPs (*N* = 11) recognised that they also lacked clarity about their roles and scope of practice. These results are reflective of other international research showing that advanced practice nursing in primary care typically focuses on health promotion, disease prevention, diagnosis, and the management of both acute and long-term health problems (Cruz Neto et al., 2023; Fagerström, 2021). However, a restrictive factor in Finland preventing NPs from working to their full scope of practice is that they are not allowed to diagnose diseases.

APNs have been shown to play an important role in improving access to meet healthcare needs (Miller et al., 2024) in domains that include family, paediatrics, women’s health, mental health, and older people’s care (Spies et al., 2024). Similarly, the Finnish NPs reported a similar variation in their expertise in seeing patients with acute and long-term health problems, such as pain, infection, abdominal and musculoskeletal problems, respiratory disease, cardiovascular disease, and diabetes—in line with other international studies (Kilpatrick et al., 2023; Torrens et al., 2020). In many countries, NPs care for and treat patients with chronic diseases in primary care (Brownwood & Lafortune, 2024). In this study, the NPs had been providing this level of care for 15 years (Glasberg et al., 2009).

The NPs in this study reported conducting follow-up assessments for patients instead of these being carried out by physicians, which increased patients’ timely access to healthcare services and reduced the workload of physicians. In addition, half of the NPs reported being responsible for patients with acute care needs despite there being limitations preventing them from working to their full scope of practice, for example, limited prescription authority and the inability to diagnose. Moreover, two of the NPs served specialised patient groups, namely child and gynaecological patients, based on their specific areas of expertise.

According to the study findings, health assessment was one of the NPs’ central practice patterns. In both Hamric’s integrative model of advanced practice nursing (Tracy et al., 2019) and the Nordic caring advanced practice nursing model (Fagerström, 2021), direct clinical practice/care was regarded as the APNs’ central competence. All NPs reported typical clinical skills for advanced nursing roles, such as physical examination and diagnostic reasoning (Schallmo et al., 2019; Spies et al., 2024). Although they could conduct diagnostic reasoning during patient consultations, the Finnish NPs felt restricted due to their inability to make a medical diagnosis in Finland; this is also the case in other Nordic countries, such as Denmark and Norway (Sulosaari et al., 2023). Diagnosis is one of the fundamental factors in fully utilising NPs’ competence and increasing their autonomy when developing their scope of practice. In the US, UK, and Ireland, there are national guidelines confirming health assessment and diagnosis as part of the NPs’ scope of practice (American Association of Nurse Practitioners, 2022; ICN, 2020; Nursing and Midwifery Board of Ireland, 2017). It is vital that diagnosis is facilitated to establish NPs’ scope of practice, enabling high-quality care to be provided to patients (ICN, 2020).

In this study, counselling and coaching for patients were viewed as core practices of advanced practice nursing, contributing to health promotion and patients’ self-care, for example, when patients seek advice after starting new medications, receiving treatment, or for weight control or diet changes. According to Audet et al. (2021), advanced practice nursing includes health condition management, lifestyle promotion (i.e. diet and physical activity), and coping strategies for patients. To meet patients’ health needs holistically, APNs use a broad range of skills, such as education, counselling, advocacy, and collaboration (Grant et al., 2017).

Another main feature of Finnish NPs’ practice patterns is the “collaborative approaches to practice,” as the participants emphasised that working with physicians who know them well enables them to work effectively. In acute care units, NPs can immediately consult physicians if the patient has complicated symptoms. In long-term health settings, NPs usually discuss patient cases with physicians once a week. Similarly, Norwegian NPs also reported a higher level of collaboration with physicians who were aware of their expertise (Kjær et al., 2025).

Additionally, the NPs in the present study mentioned their roles as consultants in patient care delivery, aligning with CNSs’ roles in improving patient care in Finland (Jokiniemi et al., 2022). Finnish NPs also collaborated with pharmacists and other professionals for patients living at home. NPs would inform pharmacists of their recommendations after a patient consultation and advise on which medications may require further consultation with a physician. To enhance patient self-care, NPs can conduct health assessments for patients at home and collaborate with the rehabilitation team to support patients’ recovery. As Torrens et al. (2020) stressed, NPs should enhance communication and collaboration as part of a multidisciplinary healthcare team in primary care.

The practice patterns of NPs remain limited in Finland due to certain restrictions. Almost half of the NPs lacked prescriptive authority, presenting a challenge for NPs in the county. The inability to diagnose diseases, in addition to limitations on prescribing to patients who have already received a physician’s diagnosis, limits the NPs’ scope of practice. In terms of nurse prescribing, the participants identified that the limitations have a negative impact on care provision in the current healthcare system, which has been echoed by others (Suutarla et al., 2023). However, it is well documented that in countries that have full prescribing rights (e.g. Ireland, the Netherlands, and the UK), patient care and access to treatment improves significantly (Maier, 2019). While countries such as the US have well-developed NP roles, NP practice in Finland is still under development. In Finland and Norway, nurse prescribing is not included in the NP programme at the master’s level (Fagerström, 2021; Sulosaari et al., 2023).

From an international perspective, leadership and research are considered two domains of APNs’ core competences (Fagerström, 2021; Tracy et al., 2019). Although the participants mentioned that they frequently used research-based national care guidelines in practice, leadership and research were not part of their practice patterns. However, Finnish NPs are skilled at transferring and implementing evidence into their practice.

A lack of clarity in the NPs’ roles and scope of practice was described as a barrier to fulfilling their advanced roles. All of the participants mentioned that they would like to have more support from organisational leaders. For example, over half of the participants wanted to have a clearer role description, which was regarded as an important factor in implementing advanced practice nursing (Josi et al., 2020; Torrens et al., 2020). Internationally, there are variations in roles, tasks, and practice structures in advanced practice nursing from country to country (Wheeler et al., 2024). Additionally, a lack of standardised education, supportive policies, and legislation often means that APNs are unable to utilise their competencies fully (Egerod et al., 2021). Despite 20 years of NP education in Finland, the participants felt that more support from healthcare organisations and clearer policy and legislation were needed for them to be able to work to their full scope of practice. Countries such as the US, the UK, and the Netherlands have clear APN policies that ensure that APNs can work to the full scope of practice (Wheeler et al., 2024). NPs are not legislated in Finland (Fagerström, 2021), which contributes to unclear practice patterns and impacts on the development of the role. In Norway, NPs are registered as Advanced Practice Generalist Nurses (Norwegian Directorate of Health, 2023); however, their roles and practice patterns are also limited and in development. Legislation, in addition to increased prescriptive and diagnostic authority, is urgently needed if NPs are to be able to work to their full scope of practice in Finland.

### Study Limitations and Strengths

Several strengths were identified in this research. First, all of the participants had worked as NPs for different patient groups, and they had diverse educational backgrounds and extensive clinical experience, which enriched the results. The use of data from interviews and open-ended questions from the survey questionnaire is another strength of this study; participants were able to provide more detailed answers in the interviews after completing the questionnaire, as they had had time to reflect on their responses. NPs who participated in this study were able to interact with each other, providing insights from diverse perspectives, during dyadic and triadic interviews; meanwhile, the individual interviews allowed for deeper and more personal insights. During the data analysis, the interpretation and abstraction levels of the data were relatively low to ensure reliability. When reporting the results, the responses from the open-ended questions and quotations from the interviews were used to support the credibility of the categories and subcategories in the results. However, the study sample was predominantly female NPs (*N* = 15), making comparative analysis unfeasible.

This study also has several limitations. The first limitation is that the NPs’ work areas varied from each other, and some participants worked with specialised patient groups, which might slightly affect the results regarding a comprehensive picture of their practice patterns. Second, this study covers only one wellbeing services county; therefore, the results may not be transferable to the entirety of Finland because the development and utilisation of advanced practice nursing varies among different regions. Nevertheless, the study is the first of its kind on NP practice patterns in primary care, and it provides new evidence that will be of interest in other Nordic countries.

## Conclusion and Recommendations

Currently, there is no reliable evidence reflecting the actual number of NPs practicing in Finland, and it is unclear to what extent NPs have been utilised at the national level. This information is essential for understanding NPs’ practice patterns across the country. This study describes common visit types to NP clinics and the main features of NPs’ practice patterns in primary care in one county, providing new insights into their roles. NPs apply their knowledge and skills in practice; however, this study clearly revealed that limitations due to prescribing and diagnostic authority restrict NPs’ scope of practice. Increased healthcare demands and prolonged waiting times for healthcare services, care, and treatment in Finland could be positively impacted by expanding the scope of practice for NPs. This should include NPs working in all areas of healthcare.

With the current healthcare reform in Finland, it is essential to consider NPs as valuable resources. It is necessary to recognise NPs’ autonomy and better utilise their competencies and resources at the regional and national levels. This could inspire more nurses to pursue further education, thereby increasing the provision of healthcare services and contributing to a sustainable healthcare environment. To align with global NP education, integrating nurse prescribing into NP education is necessary in Finland to enable an increased scope of practice and enhance the effectiveness of care provision in the Finnish healthcare system.
